# The Importance of Multifaceted Approach for Accurate and Comprehensive Evaluation of Oxidative Stress Status in Biological Systems

**DOI:** 10.3390/antiox14091083

**Published:** 2025-09-03

**Authors:** Borut Poljšak, Polona Jamnik, Irina Milisav

**Affiliations:** 1 Laboratory of Oxidative Stress Research, Faculty of Health Sciences, University of Ljubljana, Zdravstvena pot 5, 1000 Ljubljana, Slovenia; borut.poljsak@zf.uni-lj.si; 2 Department of Food Science and Technology, Biotechnical Faculty, University of Ljubljana, 1000 Ljubljana, Slovenia; 3 Institute of Pathophysiology, Faculty of Medicine, 1000 Ljubljana, Slovenia

**Keywords:** oxidative stress, reactive oxygen species, antioxidants

## Abstract

Oxidative stress is caused by an imbalance between the formation of reactive oxygen species (ROS) and the activity of antioxidant defense system, which disrupts redox signaling and causes molecular damage. While there are numerous methods to measure oxidative stress, the complex and dynamic nature of ROS production and antioxidant reactions requires a multi-faceted approach. Direct methods such as electron spin resonance (ESR) and fluorescent probes measure ROS directly but are limited by the short lifespan of certain species. Indirect methods such as lipid peroxidation markers (e.g., malondialdehyde, MDA), protein oxidation (e.g., carbonyl content), and DNA damage (e.g., 8-oxo-dG) provide information on oxidative damage, but they do not capture the real-time dynamics of ROS. The antioxidant defense system, which includes enzymatic components such as superoxide dismutase (SOD), catalase (CAT), and glutathione peroxidase (GPx), further complicates assessment, as it responds dynamically to oxidative challenges. Furthermore, the compartmentalized nature of ROS production in organelles and tissues coupled with the temporal variability of oxidative damage and repair underscores the need to integrate multiple assessment methods. This commentary highlights the limitations of using single assays and emphasizes the importance of combining complementary techniques to achieve a comprehensive assessment of oxidative stress. A multi-method approach ensures accurate identification of ROS dynamics, antioxidant responses, and the extent of oxidative damage, providing crucial insights into redox biology and its impact on health and disease.

## 1. Introduction

Oxidative stress is an imbalance between reactive oxygen species (ROS) or reactive nitrogen species (RNS) and the antioxidant defense system [1]. It is defined as “an imbalance between oxidants and antioxidants in favor of the oxidants, leading to a disruption of redox signaling and control and/or molecular damage” [2].

**Figure 1:**
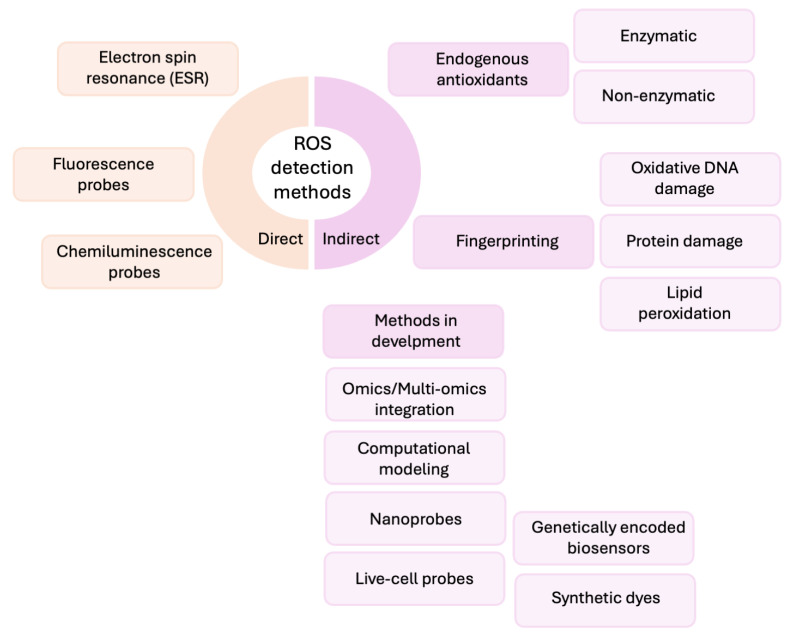
Rough classification of methods for free radicals and other reactive species detection.

The most important biologically relevant free radicals include hydroxyl (OH), superoxide (O_2_^−^), nitric oxide (NO), thiyl (RS), and peroxyl (RO_2_) species. In addition, several reactive molecules, such as peroxynitrite (ONOO^−^), hypochlorous acid (HOCl), hydrogen peroxide (H_2_O_2_), singlet oxygen (^1^O_2_), and ozone (O_3_), are not radicals themselves but can readily initiate or propagate radical reactions in the cells. The collective term reactive oxygen species (ROS) is usually used for both radical and non-radical oxidants (e.g., H_2_O_2_, ONOO^−^, ^1^O_2_, O_3_). Due to their strong chemical reactivity, ROS can interact with and damage biomolecules, making them important mediators of cell damage with potentially mutagenic and carcinogenic consequences. Since ROS are partially reduced products of oxygen, they have a high chemical reactivity with other biomacromolecules, which can lead to lipid peroxidation and oxidation of DNA, RNA, and proteins. Due to this reactivity, they play an important role in the pathogenesis of numerous degenerative and chronic diseases [3]. The superoxide anion is a primary reactive oxygen species as it can lead to the formation of other ROS such as hydrogen peroxide and hydroxyl radicals. Nitric oxide, while fulfilling important physiological functions, can react with superoxide to form peroxynitrite, a powerful oxidizing agent that can alter various biomolecules. These interactions underline the complex dynamics of oxidative stress in biological systems [4]. The interactions of ROS and RNS with organic molecules are already complex in vitro, even under simplified conditions with homogeneous solutions. In living cells, however, additional factors such as membrane surface properties, electrical charges, macromolecular binding affinities, and the spatial compartmentalization of enzymes, substrates, and catalysts further increase the complexity of these reactions [5]. In addition, free radicals have a very short half-life, which is why they are very difficult to measure in the laboratory. Today, several measurement methods are available (to detect generation of ROS or damage caused by ROS), each with their own advantages and limitations. All radicals are paramagnetic, i.e., they have one or more unpaired electrons, which creates a magnetic moment that can be studied. The measurement of free radicals poses several major challenges. Firstly, their half-life is extremely short, often only microseconds. Secondly, radicals generated in vivo usually react immediately at or very close to the site of their formation. A further complication is that many of the resulting products retain their reactivity, although usually to a lesser extent. In the clinical setting, accessible samples are generally limited to blood, urine, and breath, making direct measurement even more difficult. As a result, capturing the true biological relevance of ROS in vivo remains a considerable challenge, given the volatile and transient nature of these species. The methods for assessing oxidative stress are broadly divided into direct methods and indirect methods, with further subcategories [6,7,8]. Direct methods measure ROS levels, while indirect methods detect oxidative damage to biomolecules or evaluate antioxidant status (Figure 1). The methods developed to quantify free radicals are therefore often indirect [9,10,11].

Direct methods such as fluorescence and chemiluminescence probes, electron spin resonance (ESR), and spectrophotometric methods focus on the direct measurement of reactive species or their immediate damage products. Electron spin resonance (ESR) is a highly specific method for detecting free radicals, as it identifies unpaired electrons. Such electrons have a spin of +1/2 or –1/2, which gives them magnetic properties. In the presence of an external magnetic field, the spins align either parallel or antiparallel to the field and produce two distinct energy states that depend on the field strength. When electromagnetic radiation of the corresponding energy is applied, it is absorbed, causing the electron transition from the lower to the higher energy state. This transition leads to a characteristic absorption spectrum [12]. Direct quantification of ROS concentrations with high accuracy and precision in biological species using direct methods is difficult due to their short lifetime [13]. For this reason, indirect markers/methods for oxidative stress are used. The principle of such fingerprinting methods is to measure the products of ROS damage, i.e., not the species themselves, but the damage they cause to lipids, proteins, and DNA. For example, lipid peroxidation can be assessed using thiobarbituric acid reactive substances (TBARS), which measure malondialdehyde (MDA), a by-product of lipid peroxidation. High-performance liquid chromatography (HPLC) or gas chromatography mass spectrometry (GC-MS) are used to detect specific lipid peroxidation products such as 4-hydroxynonenal (4-HNE). Oxidation of proteins is assessed using carbonyl content assays that quantify oxidized proteins by derivatization with dinitrophenylhydrazine (DNPH). The presence of carbonyl groups in proteins serves as an indicator of ROS-mediated protein oxidation. DNA damage can be detected, for example, by measuring 8-hydroxydeoxy guanosine (8-OHdG), a biomarker for oxidative DNA damage. 8-OHdG is an important biomarker of oxidative DNA damage, as it is one of the predominant forms of DNA lesions caused by free radicals [13]. Oxidative stress results in damage to multiple biomolecules, and no single test can measure all these forms of damage simultaneously (e.g., lipid peroxidation, measured via MDA or F_2_-isoprostanes; protein oxidation, measured via protein carbonyls; and DNA oxidation, measured via 8-oxo-dG). The status of oxidative stress can also be assessed by evaluating the antioxidant defense system or related biomarkers. For example, the DPPH test (2,2-diphenyl-1-picrylhydrazyl), the ABTS test (2,2′-azinobis-(3-ethylbenzothiazoline-6-sulfonic acid) or the FRAP test (Ferric Reducing Antioxidant Power) can be used to assess the ability of biological samples to neutralize free radicals. These tests are simple and quick and are often used to evaluate the free radical scavenging capacity of natural products, biological samples, foodstuffs or other samples with a high antioxidant content. The status of oxidative stress can thus be assessed by measuring the radical-scavenging antioxidants that are consumed in the reactions with ROS. One approach is to measure individual antioxidants (e.g., ascorbate, α-tocopherol, urate, etc.) in blood, plasma, or tissue homogenates. Therefore, all individual molecules currently recognized as antioxidants should be measured [14]. However, this approach has several problems: (1) it is time-consuming, expensive and technically demanding, (2) it cannot provide information on the synergistic effects between the individual antioxidants, and (3) it may not take into account the influence of antioxidant substances that have not yet been discovered. The other approach is to measure the total antioxidant capacity (TAC) or activity by exposing the samples to controlled oxidative stress conditions and measuring either the rate of oxidation or the time to onset of oxidation. Yet another approach is to measure the activity of key endogenous antioxidant enzymes such as superoxide dismutase (SOD), which converts superoxide radicals into hydrogen peroxide, catalase (CAT), which breaks down hydrogen peroxide into water and oxygen, or glutathione peroxidase (GPx), which reduces hydrogen peroxide and lipid peroxides [15]. The evidence is based on the fact that cells under oxidative stress initiate the synthesis of endogenous antioxidants, and oxidative stress can also be analyzed by measuring specific molecules that indicate oxidative stress or damage, such as glutathione levels (GSH/GSSG ratio), which indicate the redox state of the cell. There are a number of methods to measure endogenous enzymatic antioxidants including spectrophotometric methods for measuring their activity [16,17] and molecular methods such as RT-PCR [18], DNA microarrays [19], sodium dodecyl sulfate–polyacrylamide gel electrophoresis (SDS-PAGE) with Northern blotting [20], two-dimensional electrophoresis (2-D electrophoresis) [21], and 2-D difference gel electrophoresis (2-D DIGE) [22] to measure their expression and amounts at the level of the transcriptome and proteome. From non-enzymatic systems, methods for measuring levels of glutathione [23], thioredoxin [24], and metallothioneins [25] were developed.

To summarize, direct methods focus on the measurement of reactive species. They are more immediate but often technically demanding due to the short lifetime of ROS/RNS. Indirect methods, on the other hand, provide information on the state of oxidative stress over a longer period of time by assessing antioxidants, the damage itself (fingerprinting), or damage markers. These methods are easier to perform. Measuring oxidative stress in organisms or cells poses a number of challenges due to the complexity of oxidative stress dynamics, the diversity of reactive species, and the limitations of the current methods [2,26,27]. The variability of oxidative stress markers and their dynamics in different biological contexts thus requires a multifaceted approach for an accurate and comprehensive assessment of oxidative stress status. There is no single method that can assess the overall level of oxidative stress in a biological sample (cell/tissue/organ/body). Oxidative stress can vary in space and time and can manifest itself acutely or chronically. Short-lived ROS and their downstream effects require both direct (e.g., ROS detection) and indirect (e.g., by-products of oxidative damage) methods.

Additionally, the determination of oxidative stress in cells or organs requires specialized analytical chemistry instruments that can quantify ROS/RNS, antioxidants, and markers of oxidative damage in biological samples: colorimetric or fluorometric assays, fluorescence spectrophotometers, mass spectrometry (MS) coupled with gas chromatography (GC) or high performance liquid chromatography (HPLC), electron spin resonance (ESR) spectroscopy, flow cytometry, plate readers (microplate readers), imaging systems (confocal or fluorescence microscopy), nuclear magnetic resonance (NMR) spectroscopy, and electrochemical sensors [13]. Due to the complexity and compartmentalization of oxidative stress, it is often necessary to combine several methods (e.g., HPLC-MS and fluorescence-based assays). The choice of equipment depends on the biomarkers or antioxidant enzymes of interest and the type of sample (e.g., cells, plasma, urine or tissue). The literature on methods for assessing oxidative stress is very extensive, and a comprehensive presentation is not the subject of this commentary. Rather, we will attempt to address the importance of a multifaceted approach for an accurate and comprehensive assessment of oxidative stress status.

### 1.1. Newer Methods to Assess the Status of Oxidative Stress

Newer methods such as live cell probes, genetically encoded biosensors, omics integration, computational modeling, nanotechnologies, and multiplex platforms have been reported as alternative platforms for ROS detection. Newer approaches offer complementary perspectives on oxidative processes at the molecular, cellular, and systems levels. These tools not only improve mechanistic understanding but also hold translational potential for disease diagnostics and therapeutic monitoring. Live-cell probes (small-molecule ROS-responsive fluorescent probes) are fluorescent molecular tools (either small synthetic dyes or genetically encoded sensors) that enable real-time monitoring of ROS dynamics in living cells under physiological conditions [28]. Small molecule fluorescent probes (e.g., DCFH-DA for general ROS, DHE for superoxide, mitoSOX for mitochondrial ROS) penetrate the cell, are oxidized by ROS, and generate fluorescent signals [29]. Genetically encoded probes, such as redox-sensitive GFP (roGFP), Grx1-roGFP2, and HyPer, change their fluorescence in response to oxidation and can be targeted to specific cellular compartments [30]. The advantages of such real-time monitoring are the observation of transient ROS bursts and redox changes over time within the same living cells, that high-resolution imaging allows for the identification of the precise cellular compartments (e.g., mitochondria) where oxidative events take place, and that the preservation of cell physiology avoids artifacts caused by destructive extraction methods (e.g., loss of compartment integrity, disruption of redox equilibria) [31]. Disadvantages and limitations include problems with the specificity of the probes, which can react with multiple ROS and can themselves generate ROS artifacts [32].

Genetically encoded redox/ROS biosensors are fluorescent proteins that are expressed in living cells and change their fluorescence when certain redox reactions take place. This enables compartment-resolved real-time displays [33]. It enables continuous, dynamic, reversible, and non-destructive measurement of ROS/redox status in the same cells over an extended period of time, preserving physiology and capturing transient events [28].

Omics/multi-omics integration combines data from different biological levels, such as genomics, transcriptomics, proteomics, metabolomics, and epigenomics, to obtain a holistic picture of biological responses. In the context of oxidative stress, this approach links changes in gene expression, protein content, metabolic fluxes, and regulatory markers to understand the stress pathways in detail. The integrative analysis of such data sets is made particularly difficult by the high dimensionality and heterogeneity of the data and the lack of universal analysis protocols [34]. Recent applications in oxidative stress research and the integration of multi-omics data have been presented for type 1 diabetes biomarkers [35,36].

Computational modeling has proven to be a powerful method for studying oxidative stress, allowing researchers to visualize and analyze the complex dynamics of ROS and antioxidant systems. The principle of this approach is to use mathematical frameworks such as ordinary differential equations (ODEs), Boolean networks, and Petri nets to simulate redox regulation and predict how cells respond to oxidative challenges. Dynamic ODE-based models can model continuous changes in ROS production and degradation, while discrete logic-based models are useful when kinetic parameters are not available [37,38]. These models help study the responses to oxidative stress, predict the consequences of perturbations, and provide mechanistic insights into redox regulation. One of the main advantages of computational modeling is the ability to gain mechanistic insights and predict emerging behaviors that are not easily observed experimentally. For example, models of mitochondrial metabolism have predicted how changes in membrane potential affect ROS production and redox balance [39,40]. Despite these strengths, computational models have several limitations. One major challenge is the scarcity and uncertainty of kinetic parameters, which may require assumptions that reduce accuracy [38].

Nanotechnology has provided new tools for the detection and quantification of oxidative stress, offering significant improvements over conventional biochemical assays. The principle of these methods lies in the unique physical and chemical properties of nanomaterials, such as their large surface area, tunable optical features, and catalytic activity, which can be utilized for the sensitive detection of reactive oxygen species (ROS) and associated redox processes. One approach is the use of fluorescent “switch-on” nanoprobes, which only generate a detectable signal after reacting with specific ROS, and thus offer high sensitivity and almost background-free contrast for imaging in living cells [41]. Graphene-coated plasmonic nanoprobes were used to utilize plasmon resonance energy transfer (PRET) to monitor redox-active cytochrome c in real time, enabling dynamic optical measurements of intracellular ROS such as H_2_O_2_ [42]. Another promising tool is nanoelectrodes, which can penetrate individual cells and organelles to measure ROS and reactive nitrogen species (RNS) with nanoscale resolution, revealing the spatial heterogeneity of oxidative processes [43]. The advantages of nanotechnology-based approaches include exceptional sensitivity, real-time monitoring capability, and subcellular resolution, making them powerful tools for studying dynamic oxidative processes in living systems. However, these methods are not without limitations: due to their potential nanotoxicity, engineered nanomaterials can themselves induce oxidative stress, and the complexity of nanoprobe design can pose challenges for reproducibility and translation to in vivo systems [44].

To overcome the limitations of single-parameter assays, multiplex platforms have been developed, which allow for the simultaneous detection of multiple markers of oxidative stress in a single assay. A common approach is the use of bead-based immunoassays, such as the Luminex xMAP technology, which enables parallel quantification of cytokines, oxidative stress-related proteins, and antioxidant enzymes from small sample volumes [45]. Similarly, lab-on-a-chip and microfluidic devices integrate multiple sensing elements (fluorescent, electrochemical, or colourimetric) to monitor ROS, antioxidant capacity, and biomarkers of lipid peroxidation or DNA damage under dynamic conditions [46]. In addition, multiplexed electrochemical biosensors with nanostructured electrodes have been used for the simultaneous detection of hydrogen peroxide, nitric oxide, and glutathione, enabling real-time monitoring of redox balance in biological samples [47]. The benefits of multiplex platforms include high throughput, minimal sample requirements, and a comprehensive redox profile that allows for a more holistic understanding of oxidative stress compared to single marker analyses. However, there are other challenges, including assay standardization, potential cross-reactivity between detection channels, and the need for advanced data integration tools to interpret the complex data sets generated [48]. Taken together, these innovative methods represent a paradigm shift in oxidative stress research, from static, single-parameter measurements to dynamic, multidimensional analyses. Each method brings unique strengths—from real-time imaging (live cell probes, biosensors) to comprehensive profiling (omics, multiplex platforms), predictive insights (computational modeling), and high-resolution detection (nanotechnologies). Despite certain limitations such as technical complexity, standardization issues, and potential nanotoxicity, their integration enables a more holistic understanding of redox regulation. There is still a great need to improve the current methods in order to solve the problems mentioned above.

### 1.2. Classical vs. Emerging Techniques for ROS and Oxidative Stress Detection

The classical biochemical techniques and methods, including spectrophotometric and fluorometric assays (e.g., dichlorofluorescein diacetate (DCFH-DA) for the detection of ROS, TBARS for lipid peroxidation), measurements of enzyme activity (superoxide dismutase, catalase, glutathione peroxidase), and the quantification of markers of oxidative damage, such as protein carbonyl or 8-oxo-dG in DNA [49], are relatively simple, inexpensive, and well-established, making them widely applicable. However, they are limited by low specificity, endpoint measurements, and their inability to detect dynamic or compartment-specific ROS fluctuations in living cells [50]. In contrast, new methods have been developed to overcome these limitations. Genetically encoded ROS sensors (e.g., HyPer, roGFP2-Orp1) enable real-time, ratiometric monitoring of specific ROS, such as H_2_O_2_, in different subcellular compartments, and provide unprecedented spatial and temporal resolution [51]. Nanotechnologies, including fluorescent nanoprobes and nanoelectrodes, enable highly sensitive and nanoscale detection of ROS and redox states, even at the single-cell or organelle level, although issues with nanomaterial-induced oxidative stress remain a problem [43]. Meanwhile, multiplexed platforms, such as bead-based immunoassays or lab-on-a-chip devices, enable the simultaneous assessment of multiple biomarkers of oxidative stress and antioxidant responses with minimal sample volumes, providing a more holistic view of redox balance [52]. Overall, classical methods are still valuable for initial measurements and comparative studies, especially in large-scale or resource-limited situations. However, newer approaches offer greater sensitivity, specificity, and insight into mechanisms, allowing researchers to study oxidative stress in ways that were not previously possible. The future likely lies in the integration of classical and modern techniques, combining robustness and accessibility with high resolution, real-time, and system-level perspectives.

### 1.3. Integration of Multi-Assay Data for a Coherent Oxidative Stress Status

Given the multifactorial nature of oxidative stress, a single biomarker is rarely sufficient to determine redox status. Therefore, the integration of multi-assay data is essential to capture both pro- and anti-oxidant processes. Below, we provide a brief guide to strategies for data integration, interpretation of conflicting results, and the use of bioinformatics to summarize results in a coherent framework. A meaningful assessment requires the combination of at least one marker from each category ((i) direct ROS detection, (ii) products of oxidative damage, and (iii) antioxidant defense capacity) to obtain a balanced picture of redox dynamics [53,54]. Discrepancies between the tests are not uncommon. For example, a sample may show increased antioxidant enzyme activity while at the same time showing signs of DNA oxidation. Rather than viewing these results as contradictory, they should be interpreted as reflecting different temporal or compartmental dimensions of oxidative stress. The upregulation of antioxidants may represent an adaptive response, while DNA damage may indicate that protective mechanisms were inadequate at a particular time and place [55]. New bioinformatics systems integrate data on oxidative stress with transcriptomic, proteomic, or metabolomic datasets. Network analyses can link oxidative stress markers to signaling pathways, while machine learning algorithms can classify oxidative states based on multi-assay profiles [56]. Such approaches not only improve interpretability but also enable the generation of hypotheses about upstream regulators and downstream consequences of redox imbalances. By combining conceptual frameworks, robust statistical integration, and bioinformatics tools, researchers can move beyond isolated biomarkers to a multidimensional understanding of oxidative stress. Such integrative strategies not only resolve conflicting results but also strengthen the biological relevance of findings and facilitate cross-study comparisons.

The method used should theoretically be reproducible, accurate, stable, sensitive, and capable of being used in routine clinical laboratories. Assay-specific limitations, such as specificity, sensitivity, stability, and variability by sample type, are critical for researchers when selecting methods to assess oxidative stress [57]. Any test for oxidative stress has inherent limitations that must be considered when designing studies. For example, ROS-sensitive fluorescent probes often suffer from low specificity, because many of them can react with multiple reactive species, and their stability is affected by photobleaching and sample handling [50,58]. Biomarker tests such as TBARS for lipid peroxidation are relatively sensitive, but not specific enough, as they can also detect other aldehydes in addition to malondialdehyde [59]. Protein carbonyl tests are robust but may vary depending on sample preparation and storage [60]. DNA oxidation markers such as 8-oxo-dG require careful control of artifactual oxidation during extraction and quantification [61]. Tests to determine antioxidant capacity, while useful to obtain a holistic overview, can be strongly influenced by dietary antioxidants and show variability between plasma and tissue measurements [62,63]. These test-specific limitations underline the importance of tailoring the method to the biological question and sample type and combining complementary approaches to compensate for the weaknesses of individual tests.

### 1.4. Guidelines for the Selection of Markers and Methods for Oxidative Stress

**Figure 2:**
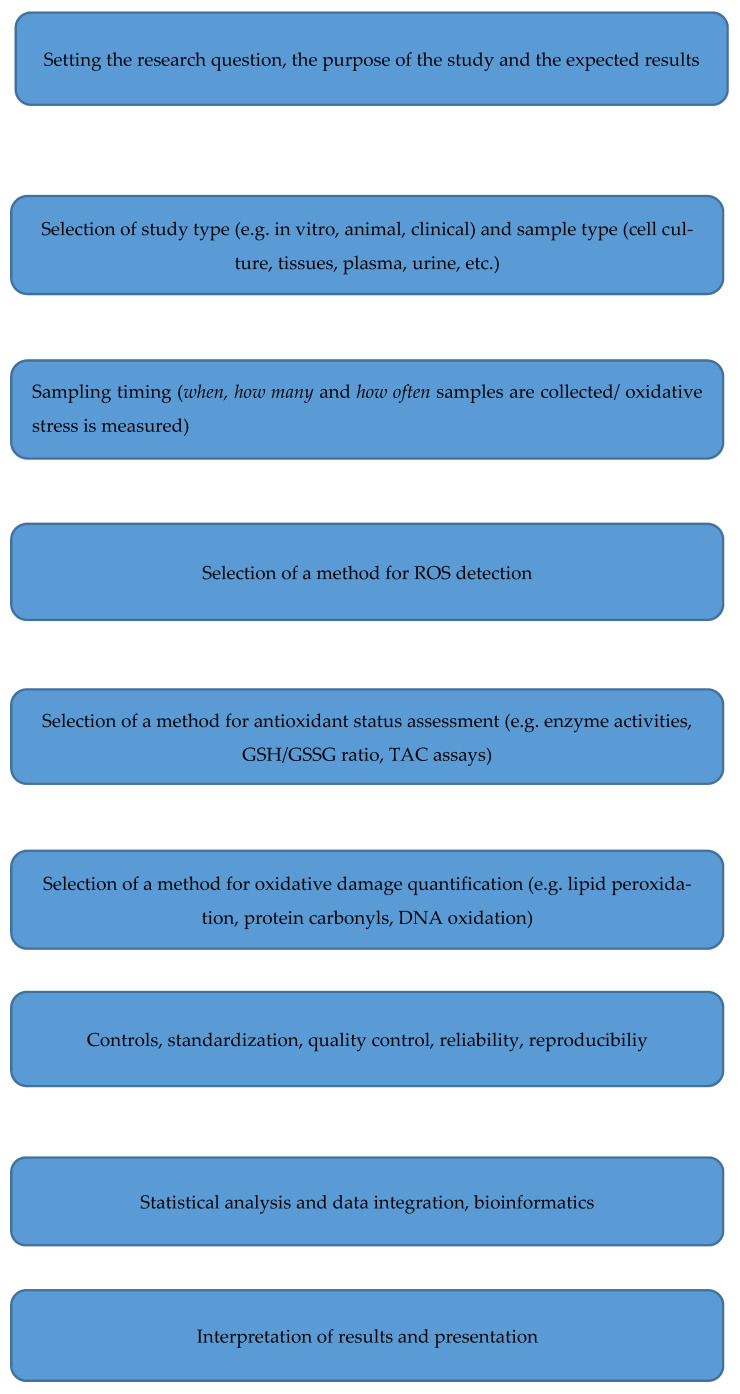
A Schematic Workflow Outlining How to Design a Multimethod Oxidative Stress Study.

Accurate assessment of oxidative stress requires careful tailoring of methodological choices to the research question, biological sample, and technical limitations (see Table 1). Since no single biomarker or assay comprehensively captures the complexity of redox biology, a structured approach is required. Defining the research objective is the first crucial step. If the goal is to study ROS dynamics or subcellular changes in real time, the use of fluorescent probes in living cells (e.g., DCFH-DA, MitoSOX) or genetically encoded biosensors (HyPer, roGFP) is most suitable due to their high spatial and temporal resolution [51,58]. In contrast, studies aiming to quantify cumulative oxidative damage or systemic oxidative burden should focus on indirect but stable markers such as lipid peroxidation products (MDA, 4-HNE, F_2_-isoprostanes), protein carbonyls, or oxidized nucleic acids (8-oxo-dG) [60,64]. To capture the adaptive side of redox biology, tests measuring antioxidant defenses, including enzyme activities (SOD, GPx, CAT), glutathione redox status, and NRF2 activation, provide valuable complementary information [54]. Sample type and stability are other important factors. While fresh samples allow direct detection of reactive species, stored samples often require the use of more stable oxidation products or antioxidant enzyme assays. At least two independent markers should be used for each target pathway, e.g., the combination of lipid peroxidation indices and DNA oxidation measurements. This approach reduces the risk of artifacts and improves interpretability. In particular, commonly used assays such as TBARS for MDA quantification have low specificity and should be complemented by more robust biomarkers such as F_2_ isoprostanes where possible [53]. The availability of resources also plays a decisive role. Laboratories with limited infrastructure can rely on spectrophotometric assays (TBARS, protein carbonyl, TAC), which, despite their limitations, enable large-scale screening at low cost. In contrast, resource-rich facilities should utilize chromatographic or mass spectrometric methods for precise and specific quantification of oxidative damage products [64]. The most informative strategy is to combine dynamic methods (ROS-sensitive probes, biosensors) with markers of cumulative damage (lipids, proteins, DNA) and assessments of defense capability (enzymes, TAC) [54,64]. This multi-layered approach ensures that the cause, consequences, and buffering capacity of oxidative stress are assessed in the specific biological or clinical setting, such as in sports physiology, where transient ROS increases play a dual role in signaling and injury [65], and in neurodegeneration, where tissue-specific oxidative stress and redox signaling are crucial [66]. Since neuronal ROS are highly compartmentalized, genetically encoded ROS sensors or live cell probes, for example, enable subcellular resolution of ROS dynamics in experimental models [51,67]. These should be combined with markers of protein oxidation (e.g., carbonyl, nitrotyrosine) and glutathione redox status in brain tissue or cerebrospinal fluid [60,68]. Such integration captures both immediate changes in redox signaling and long-term cumulative damage, which is particularly important in diseases such as Alzheimer’s or Parkinson’s [69,70]. In clinical diagnostics, direct ROS detection is rarely possible, so biomarkers of DNA oxidation (8-OHdG), lipid peroxidation (F_2_-isoprostanes) and protein oxidation (carbonyls, advanced oxidation protein products) measured in plasma or urine are the most reliable [60,71]. If these are supplemented with tests on the activity of antioxidant enzymes (e.g., superoxide dismutase, catalase) or TAC, a functional picture of the systemic oxidative balance is obtained [49,62]. Finally, strict quality control is essential for all study designs. This includes the use of non-oxidized controls, careful handling and storage of biological material, and transparent reporting of methodological details to ensure reproducibility [53]. Overall, the selection of oxidative stress markers should be guided by the biological question, the nature and stability of the samples, and the available analytical infrastructure. The use of complementary assays targeting both damage and defense mechanisms is the most robust strategy to obtain a comprehensive and biologically meaningful assessment of redox status. To summarize, a schematic workflow describing the design of a multi-method oxidative stress study is presented below (Figure 2).

### 1.5. A Schematic Workflow Outlining How to Design a Multimethod Oxidative Stress Study

**Figure 3:**
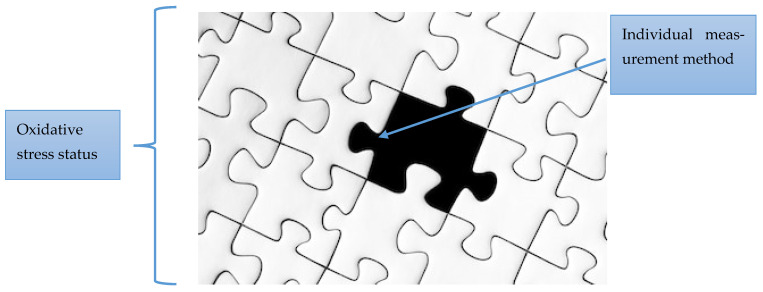
A piece of the puzzle as a single measurement compared to the whole puzzle representing the state of oxidative stress in biological systems. Each piece of the puzzle has its own role and value, but its purpose and meaning only become clear when it is connected to other pieces. Together, these pieces form a complete picture that illustrates how individual components contribute to a unified outcome.

For better understanding, we will illustrate the procedure and the importance of a multi-layered approach with the analogy of a jigsaw puzzle. Considering the current state of oxidative stress in a cell or body as analogous to a puzzle, where each piece represents a specific piece of information or evidence, misinterpreting a single piece of the puzzle can lead to a misleading or incorrect conclusion for several reasons (Figure 3). A single piece of the puzzle has no real meaning if it is not a part of the whole picture. Without the other interrelated pieces, it is impossible to see the whole picture. By focusing on just one piece and ignoring the others, one can manipulate the image and create a distorted or misleading impression. In other words, focusing on only one piece of evidence and ignoring other important parts can lead to false conclusions. In practice, this could mean using only one analytical method, analyzing only one ROS/RNS, using only one time-frame, and ignoring antioxidant defense or repair systems, among others. Possible errors resulting from the use and interpretation of just one piece of the puzzle are highlighted later in the text.

## 2. Problem of the Dynamic Nature of Oxidative Stress

Oxidative stress is a balance between constant ROS production and antioxidant defense (e.g., by enzymes such as superoxide dismutase, catalase, and non-enzymatic antioxidants such as glutathione) [72]. As such, oxidative stress in a cell or in the human body is dynamic and reflects a constant interplay between the generation of ROS and RNS and the ability of antioxidant defense systems to neutralize them (Table 2). For example, factors such as increased energy requirements, inflammation, and exposure to environmental stressors (e.g., pollution, radiation) can temporarily increase ROS production [73]. This balance is influenced by many other internal and external factors that can fluctuate over time [72]. Cells can adapt to moderate oxidative stress by upregulating antioxidant enzymes and repair pathways (Table 2). However, severe or prolonged oxidative stress can overwhelm these systems and lead to a switch from a protective, neutral to a damaging state (Table 2). Oxidative stress is not harmful per se; at low to moderate levels, it is a signaling mechanism that triggers adaptive responses, hormesis (indicated in Table 2) and survival mechanisms [74]. The physiological role of ROS in intracellular signaling and redox regulation is increasingly recognized. Nitric oxide was identified as a signaling molecule in 1987 [75] and regulates transcription factors and gene expression. Similarly, hydrogen peroxide and superoxide affect intracellular processes by oxidizing sulfhydryl-containing molecules and influencing key proteins in signal transduction and carcinogenesis [76]. ROS also mediate transcriptional control, with NF-κB and AP-1 being notable redox-sensitive transcription factors. While excessive oxidative stress is detrimental, an imbalance of cellular reductants can also disrupt redox regulation and signaling [77,78]. It should be emphasized that oxidative stress is not necessarily an undesirable situation, as its consequences can be beneficial for many physiological reactions in the cells. While antioxidants neutralize ROS to reduce oxidative stress, excessive intake, especially of synthetic antioxidants, can be harmful. This overconsumption can lead to “antioxidant stress”, a term introduced by Dundar and Aslan in 2000 [79,80]; it interferes with essential ROS-mediated physiological processes such as stress responses, pathogen defense, and systemic signaling.

This ever-changing balance of oxidants and antioxidants emphasizes the importance of time-dependent assessments in research and clinical settings to understand its role in health and disease [81]. The dynamic nature of oxidative stress therefore poses major challenges to scientists attempting to assess oxidative stress at a single time point in a cell or organism. A single time point can capture either a peak or a trough in ROS production and not reflect the average oxidative status.

### 2.1. Influence of Antioxidant Defense Mechanisms on Oxidative Stress Status

Cellular oxidative stress is not only influenced by ROS production but also by the activity of antioxidant defense systems. Endogenous antioxidant systems (e.g., catalase, superoxide dismutase, glutathione peroxidase) are adaptive by nature and can therefore be rapidly upregulated in response to oxidative stress until balance is restored. For example, cells can increase the production of antioxidant enzymes in response to increased ROS levels during exercise. This temporary upregulation ensures protection against oxidative damage during periods of increased ROS production [82]. If antioxidants have neutralized excess ROS at the time of measurement, oxidative stress may falsely appear low. On the other hand, endogenous antioxidant reactions may not immediately follow ROS production, leading to a mismatch between ROS concentrations and antioxidant activity. The induction of antioxidant enzymes may not coincide exactly with ROS production. There may be a time lag in the upregulation of antioxidant defenses following an increase in ROS, resulting in periods when ROS levels are elevated before a sufficient antioxidant response occurs. This delay can lead to transient oxidative stress that may not be detected if measurements are not taken at the right time [83].

### 2.2. Influence of Damage Repair Mechanisms on Oxidative Stress Status

An effective marker for oxidative damage should reliably increase under conditions of oxidative stress (e.g., after exposure to agents such as paraquat, diquat, ionizing radiation, or hyperoxia) and remain stable when no stress is present. Moreover, it must reflect an endogenously generated product and not one that was artifactually formed during sample preparation or isolation [84]. Cellular markers of oxidative damage are influenced not only by ROS production but also by cellular repair and damage repair systems. Differences in repair capacity between individuals or within different tissues can lead to discrepancies in oxidative damage marker values, independent of ROS production rates. For example, cells with well-functioning repair mechanisms may have lower levels of oxidative DNA lesions despite high ROS concentrations, whereas cells with impaired repair systems may accumulate damage markers even in the presence of moderate oxidative stress. This variability highlights the importance of considering repair efficiency when assessing oxidative damage and its potential biological consequences [85]. For example, the presence and level of oxidative damage markers such as 8-oxo-2′-deoxyguanosine (8-oxo-dG) in DNA are determined by the balance between the rate of oxidative damage and the capacity of repair mechanisms. Enzymes such as oxoguanine glycosylase (OGG1) recognize and remove oxidized bases and initiate repair. The efficiency of base excision repair (BER) directly affects the accumulation of oxidative lesions. For example, reduced BER activity can lead to increased levels of 8-oxo-dG, which increases the risk of mutagenesis and disease [86]. Chronic oxidative stress can overwhelm cellular mechanisms responsible for the repair or degradation of damaged lipids and proteins, such as the ubiquitin-proteasome system (UPS). During severe or prolonged oxidative stress, the UPS, which is crucial for the degradation of oxidatively damaged proteins, can be impaired. Both ubiquitin-conjugating enzymes and the proteasome itself are susceptible to inactivation by oxidative changes. This impairment reduces intracellular proteolysis, leading to an accumulation of damaged proteins [87]. High levels of ROS can trigger lipid peroxidation, which damages cell membranes and lipoproteins. This process leads to the formation of cytotoxic and mutagenic compounds such as malondialdehyde (MDA) and conjugated dienes [88]. These examples show how chronic oxidative stress can overwhelm cellular repair and degradation processes, leading to an accumulation of damaged lipids, proteins, and DNA damage over time.

### 2.3. Influence of Timing—The Importance of Choosing the Correct Measurement Interval

In order to capture the dynamic character of oxidative stress, it is crucial to adapt the measurement intervals to the kinetics of the oxidative processes. Oxidative stress is a rapidly changing state that is influenced by various cellular processes, such as the production of ROS and the activation of antioxidant defenses. These changes can occur within minutes to hours and vary depending on environmental conditions, cell type and external stimuli. For example, in a study investigating the kinetics of oxidative stress response to varying degrees of normobaric hypoxia exposure, it was found that oxidation markers increase from 30 min and peak 8 h after exposure [89]. This result underlines the need to choose appropriate measurement intervals to accurately reflect the temporal dynamics of oxidative stress. In addition, studies on the kinetics of biomarkers in patients with septic shock revealed significant fluctuations in oxidative stress markers over time, further emphasizing the importance of timing in assessment [90].

Hypoxic conditions can lead to a rapid increase in ROS levels, with a significant increase observed within 30 min to 2 h after exposure. This was shown in a study demonstrating that hypoxia induces the formation of ROS in skeletal muscle, which contributes to the stabilization of hypoxia-inducible factor-1α (HIF-1α) [91]. In contrast, antioxidant reactions, such as the activation of the Nrf2-signaling pathway, show a delayed dynamics. Research shows that 48 h of hypoxia exposure triggers Nrf2 activation and upregulates ARE-dependent antioxidant transcription in mouse skeletal muscle [92]. This underlines the importance of multiple time points to fully capture oxidative and compensatory reactions.

Biomarkers such as malondialdehyde (MDA) and F_2_-isoprostanes, which are indicators of lipid peroxidation, can fluctuate depending on the time of sample collection. These fluctuations often require measurements at multiple intervals to accurately capture the progression of oxidative stress. For example, a study examining the stability of these biomarkers in blood samples over a 36 h period found that MDA levels increased significantly over time, with intraclass correlation coefficients (ICC) below 0.1, indicating low stability. F_2_ isoprostanes also showed a significant increase with ICCs of 0.45 from 0 to 24 h and 0.09 from 0 to 36 h, indicating considerable variability [93].

As damage markers (e.g., lipid peroxidation products, 8-oxo-dG) accumulate differently over time [94], temporal variability is important in the assessment of oxidative stress, as it provides insight into the dynamic nature of ROS production, antioxidant defense responses, and resulting cellular damage. The multi-temporal approach ensures that transient peaks or troughs in oxidative markers are not overlooked, providing a comprehensive understanding of cellular redox homeostasis. Oxidative stress is not a static state; its levels can fluctuate significantly over time for the following reasons: acute stress (short-term ROS spikes) versus chronic stress (long-lasting ROS at low levels); antioxidant mechanisms, both enzymatic and non-enzymatic, respond on specific time scales to neutralize ROS; oxidative stress affects macromolecules (lipids, proteins, DNA) at different rates; and a possibly non-linear accumulation of damage markers (e.g., lipid peroxidation products) are important to understand [95].

### 2.4. Compartmentalization of Oxidative Stress

The problem of compartmentalizing oxidative stress arises from the fact that the production, reaction, and effect of ROS in the various cell compartments (e.g., mitochondria, cytoplasm, cell nucleus), tissues, and organs is very different. These differences make it difficult to take suitable samples and to assess and interpret oxidative stress, as it is not evenly distributed across the cell compartments, tissues and organs.

ROS are mainly formed in mitochondria during oxidative phosphorylation, where complexes I and III of the electron transport chain release electrons and form superoxide [96]. However, other organelles such as the peroxisomes (through fatty acid oxidation) and the endoplasmic reticulum (during protein folding) also produce ROS. It has been shown that the specific localization of ROS production determines the type and extent of oxidative damage [97]. Furthermore, the endogenous cellular antioxidants are unevenly distributed. Catalase, for example, is highly concentrated in the peroxisomes, while SOD exists in several isoforms targeting different compartments and body parts, such as Mn-SOD in the mitochondria, Cu/Zn-SOD in the cytoplasm and extracellular SOD, which can even be redistributed through the circulation [98,99]. Different tissues have different baseline ROS levels and antioxidant capacities. Tissues with a high metabolic rate, such as the brain, liver, and heart, generate significant ROS due to their high mitochondrial activity. However, their susceptibility to oxidative damage also depends on their antioxidant defenses. The brain, for example, is particularly susceptible to ROS due to its high oxygen consumption and relatively low antioxidant capacity [100].

### 2.5. Influence of Circadian Rhythms, Biological Cycles and Metabolic State on ROS Formation in Humans

The level of oxidative stress also depends on fasting, diet, sleep, physical activity, and the activity of antioxidant enzymes, which follow circadian rhythms. Mitochondrial efficiency and ROS production can be influenced by the intake of nutrients to meet energy needs, an adequate supply of micronutrients (coenzymes and vitamin-containing cofactors), calorie restriction, and moderate physical activity [1,101,102]. Fasting and calorie restriction regulate the intracellular antioxidant defense mechanisms, improve mitochondrial efficiency and reduce ROS production by activating cellular stress responses, such as the Nrf2 signaling pathway [103]. Fasting has been shown to reduce oxidative damage markers such as malondialdehyde while increasing the activity of superoxide dismutase [104]. Eating foods high in antioxidants (e.g., fruit, vegetables, and nuts) can neutralize ROS. The Mediterranean diet, for example, which is rich in antioxidants, is associated with lower oxidative stress and better cardiovascular health [105,106,107,108]. On the other hand, a diet high in processed foods, refined sugars and unhealthy fats can increase ROS production and lipid peroxidation [109,110]. Physical activity has a dual effect on oxidative stress, depending on its intensity and duration. During intense or prolonged physical activity, ROS are generated due to increased mitochondrial respiration [111], while regular and moderate exercise strengthens antioxidant defenses and reduces basal oxidative stress by upregulating enzymes such as SOD, catalase and glutathione peroxidase [112]. The body’s antioxidant systems are regulated by circadian rhythms, as the activity of antioxidant enzymes is highest at certain times of the day, reflecting the circadian regulation of cellular processes [113]. For example, SOD activity is highest during the active phase of the day to counteract increased metabolic activity [114,115]. In addition, an investigation of circadian variations in oxidative stress markers in healthy and type II diabetic subjects revealed significant diurnal effects on urinary MDA and F_2_ isoprostane levels. The study found that peak concentrations of these markers occurred in the early evening, highlighting the influence of the timing of sample collection on biomarker levels [116]. When studying oxidative stress, it is therefore important to consider the factors that influence it, such as circadian rhythm, metabolic state, healthy and harmful habits, and other factors.

## 3. Examples Showing That Using a Single Method to Assess Oxidative Stress Might Lead to False Negative or False Positive Results

Assessing the overall level of oxidative stress in biological samples is challenging because oxidative stress is a complex, multi-layered process involving a variety of ROS, RNS, antioxidant defense processes, and repair mechanisms. The reasons why no single method can comprehensively measure all oxidative stress and why relying on a single method is a significant limitation or even a mistake are outlined below. Some of the many examples are presented in the following section.

### 3.1. The Decline in Endogenous Antioxidant Enzyme Activity Could Be a Consequence of the Significantly Increased Oxidative Stress

Long-term exposure to elevated levels of ROS can impair antioxidant defense systems by directly damaging antioxidant enzymes and overwhelming their ability to maintain homeostasis. Prolonged oxidative stress causes structural and functional damage to proteins, lipids, and DNA, including antioxidant enzymes such as SOD and catalase. Over time, this leads to reduced activity of these enzymes, which further exacerbates oxidative damage [117]. Studies have shown that chronic oxidative stress associated with aging or metabolic diseases such as diabetes reduces the activity of key antioxidant enzymes through changes such as protein oxidation and enzyme inactivation. This progressive decline in antioxidant defenses contributes to a vicious cycle of increased oxidative damage and reduced enzymatic repair capacity [117,118]. These observations highlight the central role of oxidative stress in damaging the body’s antioxidant defense over time [117]. Therefore, evidence of only reduced activity of endogenous antioxidants should not generally be interpreted as a consequence of low oxidative stress.

### 3.2. The Interpretation of a Low Hydrogen Peroxide Level as an Indicator of Low Oxidative Stress Can Be Misleading

This misinterpretation arises because H_2_O_2_ concentrations reflect not only the production of ROS but also the activity of antioxidant enzymes such as catalase and SOD. Increased catalase activity, which breaks down H_2_O_2_ into water and oxygen, can lead to lower H_2_O_2_ concentrations, even under conditions of high oxidative stress [119]. Conversely, reduced SOD activity, which converts superoxide radicals into H_2_O_2_, can also lead to reduced H_2_O_2_ concentrations, masking the underlying oxidative stress [120]. A reduction in SOD activity does not directly lead to lower H_2_O_2_ concentrations, but it causes an accumulation of superoxide radicals, which can further increase oxidative stress [121]. Studies have shown that increased catalase activity often occurs as a compensatory response to increased oxidative stress, especially under conditions involving chronic ROS exposure. This adaptive mechanism efficiently reduces H_2_O_2_ concentrations, potentially leading to an underestimation of oxidative stress when H_2_O_2_ is used as only biomarker [118]. Biomarkers of oxidative stress, such as H_2_O_2_, must be interpreted in the broader context of the antioxidant defense network. Single-molecule measurements alone, such as H_2_O_2_, cannot capture the complexity of oxidative stress dynamics and can be affected by shifts in enzyme activity [117]. Therefore, the use of a combination of complementary methods—e.g., for H_2_O_2_ detection, such as ROS-specific probes (e.g., Amplex Red), together with the analysis of biomarkers (e.g., MDA, F_2_-isoprostanes) and enzyme activity tests (e.g., SOD, CAT and GPx activities)—helps minimize these errors.

### 3.3. The Dynamic Interaction of Antioxidant Enzymes (Glutathione Peroxidase, Catalase, and Peroxiredoxin) in Response to Oxidative Stress

Glutathione peroxidases and catalase both catalyze the detoxification of hydrogen peroxide, an important ROS. GPx primarily reduces H_2_O_2_ and organic hydroperoxides with the help of glutathione, while catalase converts H_2_O_2_ into water and oxygen [122]. Their cooperative function is essential for maintaining the cellular redox balance and protecting against oxidative damage. Similarly, peroxiredoxins (Prdx) neutralize H_2_O_2_ and hydroperoxides and use thioredoxin for their regeneration. In oxidative stress, catalase and peroxiredoxins can compensate for reduced GPx activity [82]. This compensation can occur with GPx inhibition or reduced substrate availability (e.g., glutathione deficiency). Misinterpretations occur because increased activity of catalase or peroxiredoxins can keep H_2_O_2_ levels low and thus mask oxidative stress. This scenario can lead to an underestimation of oxidative stress/damage and an incorrect assessment of the redox balance in biological systems. For example, catalase has a high capacity but lower affinity for H_2_O_2_ compared to GPx, making it more active at high H_2_O_2_ concentrations, while Prdxs play a key role in signaling by selectively reducing certain peroxides [2,123]. In summary, a combination of methods should be used to analyze the conversion of the H_2_O_2_ molecule.

### 3.4. Relying on a Single Biomarker, Such as Glutathione (GSH) Levels, to Assess Oxidative Stress Can Lead to Misinterpretation Due to the Complexity and Compensatory Nature of the Redox System

Glutathione occurs in two forms: reduced (GSH) and oxidized (GSSG). The ratio of GSH to GSSG is often used as a measure of oxidative stress. However, enzymes such as glutathione reductase (GR) can convert GSSG back into GSH and thus keep the GSH levels high even in the event of oxidative damage. This compensation can mask oxidative stress when GSH levels are measured in isolation [123]. Elevated GSH levels are therefore not necessarily an indication of low oxidative stress but may result from increased activity of enzymes such as thioredoxin reductase (TrxR) or GR, which replenish the reduced forms of antioxidants in response to oxidative stress. This compensatory mechanism can mask ongoing oxidative damage [7,17,124]. A multi-marker approach should be pursued in which the GSH concentration is considered together with GSSG, enzyme activities (e.g., GR, TrxR), and other markers of oxidative stress.

### 3.5. Low Oxidative Stress Could Be the Result of Low ROS Formation or High ROS Formation Combined with Increased Antioxidant Defenses

The extent of oxidative stress in a biological system is not determined solely by the ROS content but by the balance between ROS production and the activity of antioxidant systems. Low oxidative stress can occur when ROS production is minimized due to reduced metabolic activity, suppressed inflammatory responses, or upregulation of ROS-scavenging enzymes such as SOD and CAT. For example, cells under hypometabolic conditions exhibit lower ROS production and oxidative stress, as found in studies on caloric restriction and hypoxia [125,126]. Even under conditions of increased ROS production, oxidative stress can remain low if the antioxidant system efficiently neutralizes the ROS. Increased activity of enzymatic antioxidants (e.g., SOD, CAT and GPx) and non-enzymatic antioxidants (e.g., glutathione, vitamins C and E) can prevent the accumulation and damage of ROS. Studies have shown that cells exposed to moderate oxidative challenges often adapt by upregulating antioxidant defenses and achieving redox homeostasis [127,128]. This adaptive response improves the system’s ability to counteract ROS and keeps oxidative stress at a low level even when ROS formation is relatively high [129,130].

### 3.6. Increased Activity of Cellular Repair Systems Can Effectively Counteract Oxidative Damage, Resulting in Low Levels of Detectable Damage Even Under Conditions of Significant Oxidative Stress

Cells have robust DNA repair pathways such as base excision repair (BER), nucleotide excision repair (NER), and mismatch repair (MMR). These systems actively repair oxidative DNA lesions such as 8-oxoguanine, a common marker of oxidative DNA damage. Increased activity of these pathways can maintain low levels of detectable DNA damage even in environments of increased oxidative stress [131]. Oxidatively damaged proteins can be repaired by enzymatic mechanisms such as protein disulfide isomerases or selectively degraded by the proteasome system. For example, the thioredoxin and glutaredoxin systems play a crucial role in the repair of oxidized cysteine residues and mask oxidative protein damage [132,133,134]. Repair systems such as Prdx and enzymes such as phospholipase A2 and GPx can detoxify lipid hydroperoxides and thus reduce the concentrations of detectable lipid peroxidation markers despite considerable oxidative challenges [135,136,137]. In cases where the repair systems are activated and oxidative stress is not high enough to affect the repair systems, measuring only one parameter of oxidative stress-induced damage can be misleading.

### 3.7. The Widely Used Dichlorofluorescein Test Measures the General ROS Content, but Due to Its Slower Reaction Kinetics, It Can Overlook Short-Lived Species Such as Superoxide or Hydroxyl Radicals, So the Test Results Can Falsely Imply Low Oxidative Stress

The dichlorofluorescein (DCF) assay uses the fluorescence of dichlorodihydrofluorescein (DCFH), which is oxidized to DCF in the presence of ROS [138]. Although the test is widely used due to its relative simplicity and broad applicability, it has one major limitation, namely the detection of specific ROS. The test determines general ROS values but does not differentiate between different ROS [139]. It primarily detects H_2_O_2_ and other ROS with a relatively long half-life. Short-lived ROS such as superoxide and hydroxyl radicals, which have very fast reaction rates and lifetimes in the order of nanoseconds to microseconds, may not be efficiently detected using the DCF assay [50]. This is because DCFH, the precursor of DCF, requires an oxidative species with a longer residence time to interact sufficiently to produce measurable fluorescence. Superoxide, for example, is rapidly converted to hydrogen peroxide by SOD, making its direct detection by the DCF assay difficult. In addition, hydroxyl radicals are extremely reactive and short-lived, often causing significant cell damage, but cannot be detected by DCF due to their rapid reactivity. As a result, the test could potentially underestimate oxidative stress in systems where these short-lived radicals are prevalent. To overcome this limitation, various assays/methods such as cytochrome c reduction assays for superoxide [140] or ESR spectroscopy for the detection of hydroxyl radicals [141] are used.

### 3.8. Malondialdehyde Is Commonly Used as a Lipid Peroxidation Marker, but Other Aldehydes or Unrelated Substances May React with the Test Reagents, Resulting in Overestimation

MDA is widely known as a by-product of polyunsaturated fatty acid peroxidation and is commonly used as an indirect marker of oxidative stress and lipid peroxidation. It is measured by reaction with thiobarbituric acid in the TBARS (thiobarbituric acid reactive substances) assay, producing a colored product. However, 4-hydroxy-2-nonenal (HNE), malonaldehyde, and other reactive aldehydes can also be formed during lipid peroxidation, which give similar products in the TBA assay and can falsify the measurement [142]. For example, acrolein and ethylmalondialdehyde can also react with the TBA reagent to produce colorimetric products that can be misinterpreted as MDA. These aldehydes can be formed in various ways that have nothing to do with lipid peroxidation. In addition to lipid-derived aldehydes, glucose metabolism and protein oxidation can lead to the formation of aldehydes such as glyoxal and methylglyoxal, which can interfere with the MDA assay and contribute to an overestimation of lipid peroxidation values. Several other compounds can also generate reactive intermediates that react with TBA, including carbonylated proteins and sugars [26]. This leads to false-positive or inflated MDA values, which can mask the true extent of lipid peroxidation. For a more specific and sensitive detection of MDA and other lipid peroxidation products, allowing them to avoid the cross-reactivity observed with the TBARS test, liquid chromatography-mass spectrometry (LC-MS) [143], or HPLC [144] techniques should be additionally used.

### 3.9. The Carbonyl Content of Proteins Can Increase Due to Aging or Other Non-Oxidative Changes Such as Glycation and Can Be Interpreted as a False Indication of Oxidative Stress When Oxidative Damage Is Not the Actual Cause

ROS can directly oxidize amino acid side chains such as proline, arginine, lysine and threonine, leading to carbonyl formation. These modifications are often used as indicators of oxidative stress in various biological systems [145,146]. However, the carbonylation of proteins is not exclusively caused by ROS-mediated oxidation. Non-oxidative processes such as glycation (the reaction of reducing sugars or dicarbonyl compounds such as methylglyoxal with protein amino groups) can also generate carbonyl groups. The advanced glycation end products (AGE) formed by glycation accumulate with increasing age or diabetes and lead to an increased carbonyl content in proteins independently of oxidative stress [147,148]. For example, increased protein carbonyl levels in aging or diabetic patients could be incorrectly interpreted as oxidative stress. Furthermore, in aging cells, impaired proteostasis and reduced proteasome activity can lead to the accumulation of modified proteins, regardless of the degree of oxidative stress [149,150]. Glycation and oxidation pathways can sometimes overlap, further complicating interpretation. Therefore, using protein carbonyl content as the sole marker of oxidative stress in clinical studies may lead to misleading conclusions, especially in conditions such as diabetes where glycation predominates.

## 4. Limitations

With our commentary we wish to emphasize the importance of a multi-faceted approach to the assessment of oxidative stress, while being aware of certain limitations. Given the scope and complexity of this field of research, it was not possible to provide an exhaustive overview of all relevant methods, markers, and analytical frameworks. Therefore, some important aspects could only be briefly addressed. For example, several well-established methods exist to control and study specific ROS-mediated signaling pathways. These include pathways regulated by transcription factors such as NRF2, which not only protect normal cells, but can also promote the survival of malignant cells and thus contribute to the development and progression of various cancers and non-cancerous diseases [151,152]. Another example is NF-κB, where excessive or prolonged activation by ROS drives chronic inflammation and is implicated in the pathogenesis of cancer, neurodegeneration, cardiovascular diseases, and metabolic disorders [153,154]. Some markers were omitted for clarity, although they may also be important, such as the enzyme paraoxonase-2, which detoxifies ROS and is upregulated in many cancers [155,156,157]. In addition, the field of statistics was omitted, which has a significant impact on summarizing the results into a coherent oxidative status. To provide clarity and maintain focus on the central message of the manuscript, we deliberately emphasized certain topics while acknowledging that additional perspectives may be valuable for a comprehensive understanding of the biology of oxidative stress.

## 5. Conclusions

The increasing interest in the role of oxidative stress in disease pathogenesis has led to an increased need for techniques to measure ROS/RNS and their responses in vivo and especially in clinical situations. A variety of analytical methods have been developed to measure different end products of ROS reactions with cellular components, but not all of them are applicable in clinical situations where usually only blood, urine, and breath samples are available. Furthermore, it is not possible for a reactive radical generated in an internal tissue—with a lifetime of only microseconds—to diffuse into the blood or other fluids so that it can be detected at a remote location. The researcher is therefore strictly limited to determining secondary products in body fluids remote from the site of the original damage. Advances in understanding the effects of treatment on oxidative damage in human disease will be greatly facilitated if several biomarkers of oxidative damage are routinely included in clinical trials of therapeutic agents. This manuscript emphasizes the interdependence of the individual elements (e.g., measurement method) in establishing a coherent overall status of oxidative stress. Given the complexity of oxidative stress, no single method can provide a complete picture. For example, a study by Halliwell and Gutteridge (2015) [26] showed that ESR spectroscopy is effective for detecting free radicals in vitro but does not provide accurate measurements in complex biological samples. Conversely, lipid peroxidation assays such as TBARS are excellent for quantifying oxidative damage over time but not as good for measuring ROS fluctuations in real-time, emphasizing the need for a combined approach. A combination of methods enables a nuanced understanding of ROS dynamics, the extent of biomolecular damage, and the efficacy of antioxidant defenses, leading to a better understanding of cell health, cellular oxidation, and disease mechanisms. To overcome the limitations described in the manuscript, several complementary methods should be used to comprehensively assess oxidative stress at different time intervals. Until more robust methods are available, it is generally recommended to use at least two complementary assays, as each method captures different aspects of oxidative stress and has its own limitations; no single technique alone can provide accurate quantification of ROS. Such an approach would allow the detection of the full spectrum of ROS and damage markers, the assessment of enzymatic and non-enzymatic antioxidant defenses, the localization of oxidative stress in specific tissues or cellular compartments, and the assessment of the temporal profile of oxidative stress and its dynamics. Strategies to overcome the challenges described in the manuscript should be recognized and addressed accordingly to improve the reliability and accuracy of oxidative stress assessment by using real-time measurement tools that assess ROS concentrations, antioxidant activity, and damage repair. This can be achieved by combining static and dynamic assessments—for example, tracking biomarkers of oxidative stress over time while accounting for circadian rhythms and external influences (such as diet, physical activity, etc.). There is an urgent need for a critical evaluation of the information that each test can provide to better demonstrate the applicability and limitations of the results on specific samples and to justify the choice of one method over another. To advance the field, future research should focus on several key directions. First, there is a need for standardized protocols and reference materials that allow cross-study comparison and reproducibility of oxidative stress measurements. Second, advances in real-time biosensor technology, including genetically encoded probes, nanotechnology sensors, and multiplex platforms, should be further developed and validated for both experimental and clinical purposes. Third, the integration of multi-omics approaches with computational modeling and bioinformatics will be crucial to merge the different data streams into a coherent profile of oxidative stress. Finally, closer collaboration between basic researchers, technology developers, and clinicians is needed to translate methodological advances into clinically useful diagnostic and therapeutic approaches.

## Figures and Tables

**Table 1:** Comparison of commonly used techniques to assess oxidative stress.

***Direct Methods (Measure ROS or Oxidation Products Directly)***				
**Method**	**Principle**	**Advantages**	**Limitations**	**Sample type**
**ESR (Electron Spin Resonance)**	Direct detection of unpaired electron species (free radicals).	Standard method for direct ROS detection; high specificity.	Low sensitivity in biological samples; requires specialized equipment; limited clinical use.	In vitro samples, cell systems
**ROS-sensitive fluorescent probes (e.g., DCFH-DA, MitoSOX)**	Fluorescent dye oxidation proportional to ROS levels in live cells.	Real-time detection; dynamic monitoring; applicable in live-cell imaging.	Non-specific for ROS type (react with multiple ROS/RNS); prone to artifacts from light and redox cycling; variability in uptake/stability.	Cells, tissues
**Genetically encoded ROS sensors**	Reporter proteins (e.g., roGFP, HyPer) that change their fluorescence upon oxidation.	High specificity; allows compartment-specific, real-time ROS monitoring.	Requires genetic manipulation; limited applicability in clinical samples; technical expertise needed.	Cells, tissues (in vitro/in vivo models)
***Indirect methods (measure damage or antioxidant defense)***				
**Method**	**Principle**	**Advantages**	**Limitations**	**Sample type**
**TBARS (Thiobarbituric Acid Reactive Substances)**	Measures malondialdehyde (MDA), a lipid peroxidation byproduct.	Simple, inexpensive, widely used.	Low specificity (interfering substances, other aldehydes detected); artifacts during sample handling, indirect measure of ROS.	Plasma, serum, tissues
**Protein Carbonyl Assay**	Detects carbonyl groups introduced into proteins by oxidative modification.	Good marker of protein oxidation; relatively stable products.	Lacks information on specific proteins involved; not real-time; may underestimate reversible oxidative modifications.	Plasma, serum, tissues, extracts
**8-oxo-dG (DNA oxidation marker)**	Quantifies oxidized guanine in DNA via HPLC, ELISA, or MS-based techniques.	Specific biomarker for oxidative DNA damage.	Requires careful sample handling to prevent artifacts; expensive if MS-based; susceptible to artefactual oxidation during isolation.	DNA from tissues, blood, urine (as excreted metabolite)
**Antioxidant enzyme activity (e.g., SOD, CAT, GPx)**	Measures enzymatic defense capacity against ROS.	Provides insight into endogenous defense status.	Indirect; enzyme activity may vary due to unrelated factors (e.g., circadian rhythm, diet).	Plasma, serum, tissues
**Glutathione (GSH/GSSG ratio)**	Assesses redox balance by quantifying reduced vs. oxidized glutathione.	Sensitive measure of redox status; widely validated.	Requires rapid sample processing; GSH is easily oxidized ex vivo.	Blood, tissues, cells
**Total antioxidant capacity assay**	Cumulative action of all antioxidants in a sample	Offering a simple assessment of antioxidant potential	Cannot distinguish contributions of individual antioxidants; influenced by sample matrix effects and diet; results vary across assays (FRAP, ORAC, etc.)	Plasma, serum, saliva, or urine

**Table 2:** Challenges in collecting and analyzing biological samples over a time period, the extent of oxidative stress formation, and the opposing activity of antioxidant protection and damage repair systems stimulated by the ROS triggering of hormesis (* indicates the triggering of hormesis).

Grade of Oxidative Stress Generation from Exogenous or Endogenous Sources	Level of Intracellular ROS	Oxidative Damage	Activity of Endogenous Antioxidant Enzymes	Activity of Damage Removal Systems	Appropriate Markers	Detection Methods
Low	Low	No	Slightly increased *	None	Basal levels of reactive oxygen species (ROS) (e.g., superoxide, hydrogen peroxide). Reduced glutathione (GSH) levels. NADPH/NADP+ ratio (indicating redox homeostasis). Subtle changes in mitochondrial membrane potential.	Direct measurement of ROS by spectroscopic technique of electron spin resonance (ESR)/electron paramagnetic resonance (EPR) or pulse radiolisis. Estimation of intracellular oxidation with fluorescent probes (e.g., dihydroethidium (DHE) and Mito SOX red for superoxide and Amplex Red for hydrogen peroxide). Measuring changes in endogenous antioxidant enzymatic and non-enzymatic defense systems. Measuring changes in intracellular redox state sensors. Mitochondrial activity indicators (e.g., JC-1 or TMRE for membrane potential changes).
Moderate	Mild * to moderate	Some oxidative damage	No further increase in the activity of endogenous antioxidant enzymes	Low* to moderate activity of endogenous oxidative damage repair mechanisms	Increased ROS levels (e.g., mitochondrial and cytosolic ROS).	Direct measurement of ROS by electron spin resonance (ESR)/electron paramagnetic resonance (EPR) or pulse radiolysis. Estimation of intracellular oxidation with fluorescent probes. Measuring changes in endogenous antioxidant enzymatic and non-enzymatic defense systems.
High	High	Moderate oxidative damage	Reduced activity of endogenous antioxidant enzymes (inactivation, denaturation)	High activity of endogenous oxidative damage repair mechanisms *	Mild to moderate oxidative damage to lipids (e.g., lipid peroxidation by-products (e.g., malondialdehyde, MDA), proteins (protein carbonylation), and DNA (oxidized nucleotides (e.g., 8-oxo-dG)).	Direct measurement of ROS by spectroscopic technique of electron spin resonance (ESR)/electron paramagnetic resonance (EPR) or pulse radiolysis. Estimation of intracellular oxidation with fluorescent probes. Fingerprinting methods of oxidative DNA, protein damage and lipid peroxidation products.
Severe	Extreme (chronic oxidative stress)	Presence of severe oxidative damage	The activity of endogenous antioxidant enzymes decreases due to their own damage (denaturation)	Endogenous oxidative damage repair mechanisms suppression	Significant depletion of antioxidant defenses (e.g., GSH, superoxide dismutase, catalase). Substantial oxidative damage to biomolecules. Dysregulated mitochondrial function (e.g., mitochondrial DNA, loss of membrane potential, release of cytochrome c). Extreme ROS levels leading to cell death (apoptosis or necrosis). High levels of inflammatory markers linked to oxidative damage (e.g., NF-κB activation).	Direct measurement of ROS by spectroscopic technique of electron spin resonance (ESR)/electron paramagnetic resonance (EPR) or pulse radiolisis. Estimation of intracellular oxidation with fluorescent probes. Fingerprinting methods of oxidative DNA, protein damage and lipid peroxidation products. Antioxidant enzyme assays. Mitochondrial assays. Cell viability and death assays. Inflammatory markers (e.g., TNF-α, IL-6).
